# Feasibility, safety, and acute efficacy of the fourth‐generation cryoballoon for ablation of atrial fibrillation: Another step forward?

**DOI:** 10.1002/clc.23328

**Published:** 2020-02-25

**Authors:** Laura Rottner, Shibu Mathew, Bruno Reissmann, Laura Warneke, Isabell Martin, Christine Lemes, Tilman Maurer, Peter Wohlmuth, Feifan Ouyang, Karl‐Heinz Kuck, Andreas Metzner, Andreas Rillig

**Affiliations:** ^1^ Department of Cardiology Asklepios Klinik St. Georg Hamburg Germany; ^2^ Department of Cardiology Asklepios Klinik Harburg Hamburg Germany; ^3^ Asklepios Proresearch Asklepios Klinik St. Georg Hamburg Germany; ^4^ Department of Cardiology Universitäres Herzzentrum Hamburg Eppendorf Hamburg Germany

**Keywords:** atrial fibrillation, catheter ablation, cryoballoon

## Abstract

**Background:**

The second‐generation cryoballoon (CB2) is widely used for pulmonary vein (PV) isolation (PVI) in patients with paroxysmal atrial fibrillation (AF). Recently, the novel fourth‐generation CB (CB‐Advance PRO) was introduced, incorporating a shortened catheter tip.

**Hypothesis:**

The aim of this study was to evaluate the feasibility and acute efficacy of PVI using the CB‐Advance PRO.

**Methods:**

A total of 200 consecutive patients were analyzed. Hundred patients who underwent PVI due to symptomatic, drug‐refractory AF were treated with the CB‐Advance PRO (group I) and were included into this multicenter analysis. A group of 100 patients were treated with the CB2 and acted as controls (group II).

**Results:**

In total, 739 of 739 PVs (100%) were successfully isolated. There was a nonsignificant trend in the incidence of online registration of PV signals between both groups (group I: 77.9% vs group II: 71.4%, *P* = .09). Median time to PVI (time to isolation [TTI]) and mean total freezing time were significantly shorter when using the CB‐Advance PRO (group I: 33 [23, 50] vs group II: 40 [26, 60] seconds and group I: 166 ± 29 vs group II: 183 ± 38 seconds, *P* < .01). In three of 100 (3%) patients of group I and one of 100 (1%) patients of group II, a transient phrenic nerve palsy occurred (*P* = .62).

**Conclusion:**

The use of the novel CB‐Advance PRO is feasible and associated with a significant reduction in mean TTI and mean total freezing time as compared to the CB2.

## 1 INTRODUCTION

Atrial fibrillation (AF) is the most common sustained cardiac arrhythmia. Maintenance of stable sinus rhythm is intended in symptomatic patients, and catheter ablation aiming at pulmonary vein (PV) isolation (PVI) provides the most effective treatment option with encouraging clinical outcome data.^1^, ^2^, ^3^ Different energy sources and various ablation tools have been evaluated to improve the success rate of PVI.^4^, ^5^, ^6^, ^7^ The FIRE AND ICE trial, which prospectively randomized patients with paroxysmal AF (PAF) to either radiofrequency (RF) or cryoballoon (CB)‐based PVI, demonstrated noninferiority of CB ablation as compared to RF ablation in terms of efficacy and safety.^6^ CB‐based AF ablation is currently the only established “single‐shot” ablation modality and is not only readily applicable and easy to learn, but also a particularly safe tool.^1^, ^8^

CB ablation strategies may apply fixed freeze cycle durations of 180 to 240 seconds. Recently, ablation protocols were modified for shorter and fewer CB applications aiming at reduction of periprocedural complications such as phrenic nerve (PN) palsy.^8^ Therefore, the individual time to isolation (TTI) has been implemented which resulted in shorter procedure times dependent on the bonus freeze strategy but comparable clinical outcome.^9^ The length of the distal tip of the second‐generation CB (CB2) might limit optimal visualization of PV signals in some patients as it might prevent proximal positioning of the circular mapping catheter (Achieve; Medtronic) within the PV.^10^

**Figure 1:**
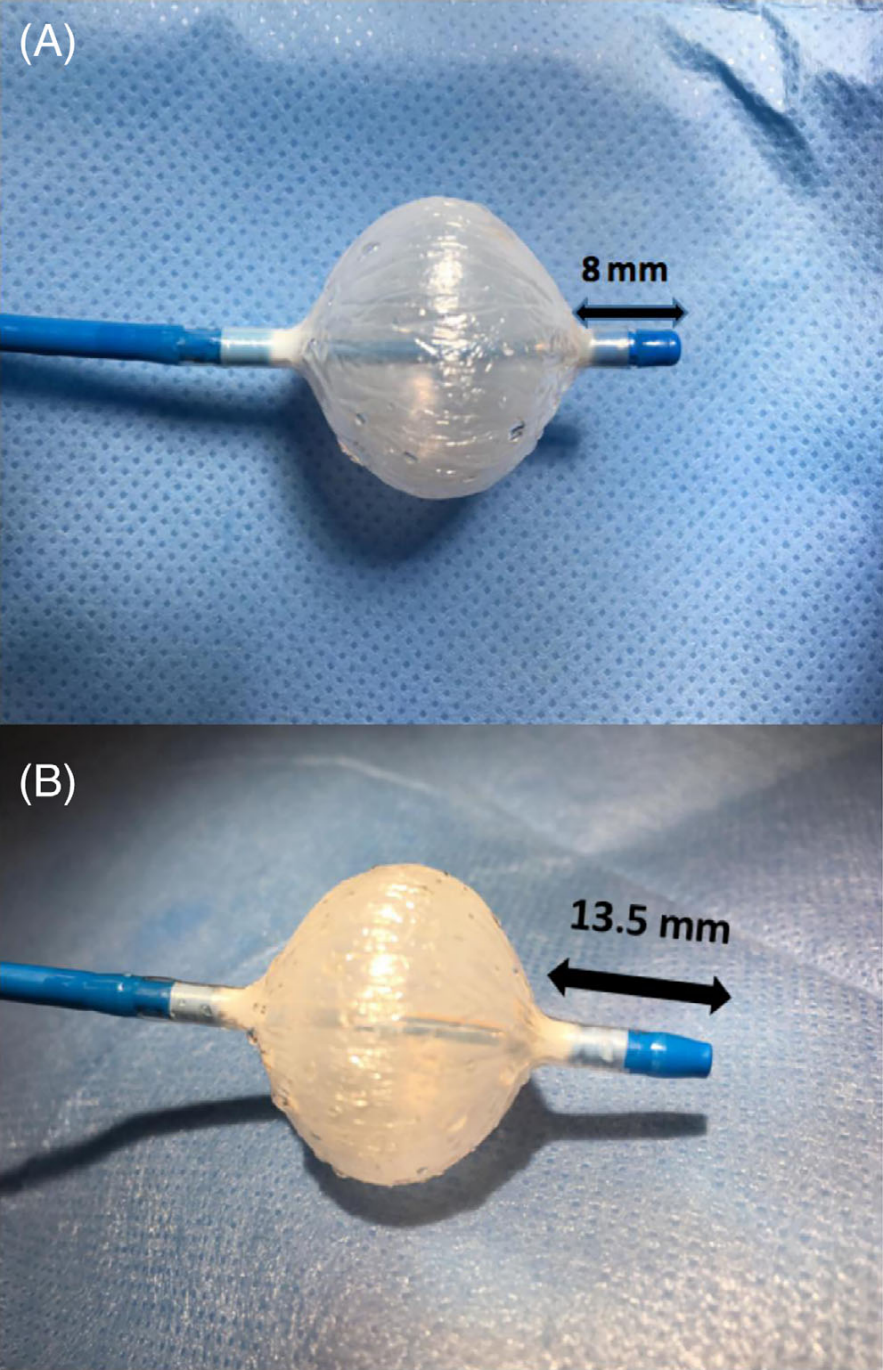
Novel Arctic Front Advance Pro (Medtronic, Inc., Minneapolis, Minnesota) with a shorter balloon tip (8 mm). CB2 in comparison. CB2, second‐generation cryoballoon

**Figure 2:**
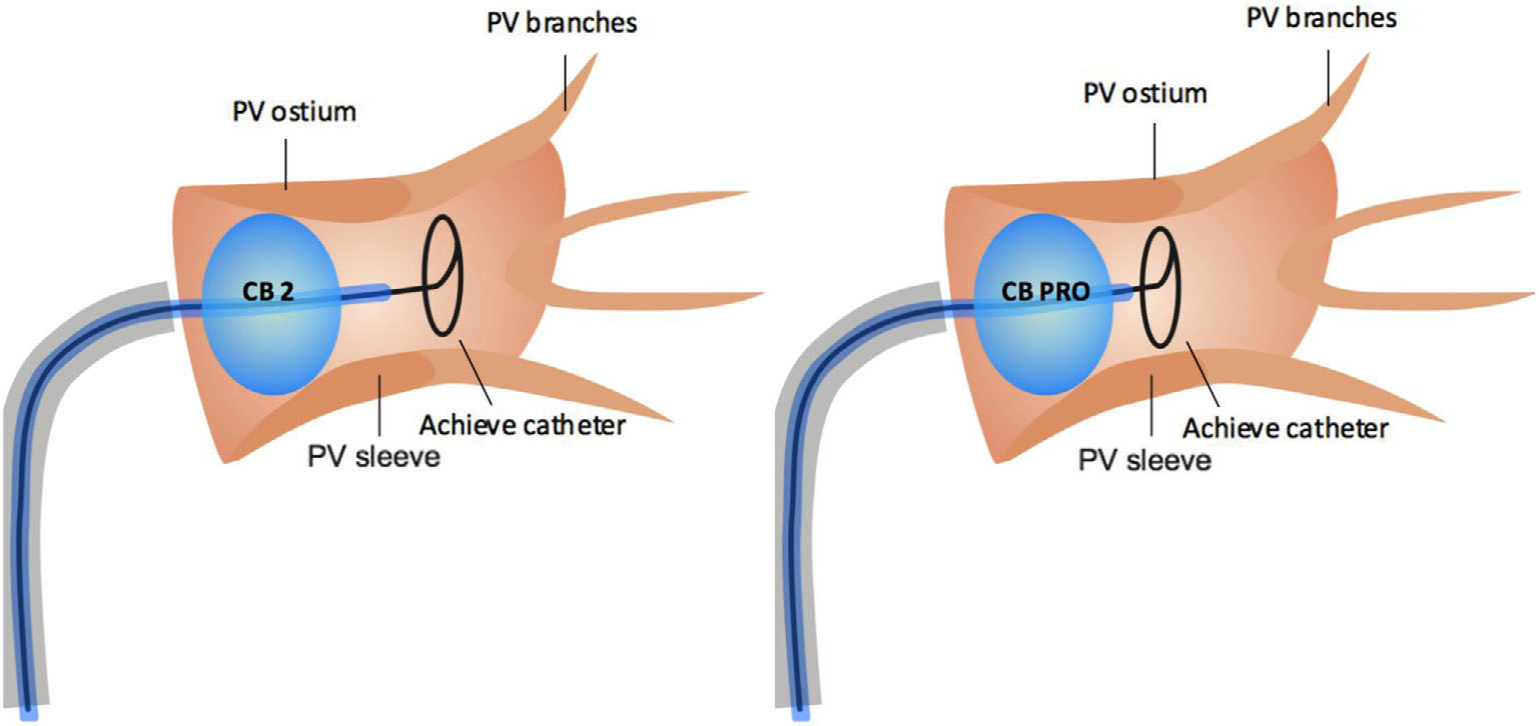
The 40% shortened tip of the CB‐Advance PRO in comparison to the CB2 is demonstrated and the possibility of a favorable positioning of the Achieve catheter toward the PV ostium/sleeves is emphasized. CB, cryoballoon; CB2, second‐generation CB; CB‐Advance PRO, fourth‐generation CB; PV, pulmonary vein

Recently, the novel fourth‐generation CB (CB‐Advance PRO, Artic Front Advance Pro; Figure 1) was introduced into clinical practice, featuring a 40% shortened tip to improve catheter maneuverability and visualization of TTI (Figure 2). This design was considered to translate into an increased rate of PV real‐time signal recording, thus facilitating individualized ablation strategies.

The purpose of this retrospective analysis was to evaluate the feasibility and acute efficacy of PVI using the CB‐Advance PRO as compared to the CB2.

## 2 METHODS

### 2.1 Study design and patient population

In total, 100 consecutive patients suffering from symptomatic, drug‐refractory PAF or persistent AF who underwent first‐time PVI using the novel CB‐Advance PRO at the two experienced clinics (Asklepios Klinik St. Georg or Asklepios Klinik Harburg in Hamburg, Germany) were included into this study (group I). A control group of 100 consecutive patients with paroxysmal or persistent symptomatic, drug‐refractory AF who underwent de novo PVI using the CB2 served as control (group II). Exclusion criteria were prior PVI, contraindications to post‐interventional oral anticoagulation, and severe valvular heart disease. The study was approved by the local ethics board (WF‐05/19) and was performed in accordance with the Declaration of Helsinki.

### 2.2 Preprocedural preparation

Transesophageal echocardiography was performed before the ablation procedure to rule out intracardiac thrombus formation in all patients. In patients on vitamin K antagonists, ablation was performed under therapeutic international normalized ratio (INR) values of 2 to 3. Direct oral anticoagulants were stopped the day before the procedure and re‐initiated 6 hours thereafter.^11^

### 2.3 AF ablation procedure

CB‐based ablation was only performed by highly experienced CB operators based on individual operation volume as suggested in current guidelines.^1^ Ablation procedures were performed according to our routine protocol as previously described.^8^ In brief, single transseptal puncture was performed using a modified Brockenbrough technique and an 8.5‐Fr transseptal sheath (SL1; St. Jude Medical, St. Paul, Minnesota). After transseptal puncture, a heparin bolus was administered targeting an activated clotting time >300 seconds. Selective PV angiographies were performed to identify the individual PV ostia. The transseptal sheath was exchanged over a wire for a 12‐Fr steerable transseptal sheath (Flexcath Advance; Medtronic). A temperature probe (Sensitherm, St. Jude Medical or Circa S‐Cath, Circa Scientific, Englewood, Colorado) was placed within the esophagus to monitor esophageal temperatures during freeze cycles (cutoff value: 15°C). In all procedures, a 28‐mm CB was used. During CB applications along the septal PVs, continuous pacing of the PN was performed via a diagnostic catheter positioned within the superior caval vein (7 Fr, Webster; Biosense Webster, Diamond Bar, California). Pacing was set at maximum output and pulse width, and a cycle length of 1000 ms. Monitoring of PN was based on tactile feedback of diaphragmatic contraction and on assessment of the right diaphragmatic compound motor action potential (CMAP). Energy delivery was interrupted immediately if weakening or loss of diaphragmatic contraction was noted or a decrease of the CMAP amplitude ≥30% was observed. The CB was advanced into the left atrial (LA) over a spiral mapping catheter (Achieve; Medtronic). The CB was inflated proximal to the PV ostium. Complete PV occlusion was verified by contrast medium injection through the central lumen of the CB. In all procedures, our protocol aimed at a TTI‐guided ablation strategy based on continuous real‐time recordings from the circular mapping catheter as reported previously.^4^ After documentation of PVI, the freeze cycle was prolonged for additional 120 seconds. If no real‐time PV signal recording could be obtained, a standard freeze cycle of 180 seconds was applied. No bonus freeze cycle was applied.

Procedural endpoint was defined as persistent PVI verified by spiral mapping catheter recordings.

### 2.4 Postprocedural care

Following the ablation procedure, all patients underwent transthoracic echocardiography to rule out pericardial effusion. All patients were treated with proton‐pump inhibitors for 6 weeks. Anticoagulation was continued for at least 3 months post ablation and then based on the individual CHA_2_DS_2_‐VASc score. Previously ineffective antiarrhythmic drugs were recommended for 3 months.

### 2.5 Endpoints

The primary endpoint was the incidence of real‐time recordings and electrical isolation of the PVs as assessed by a circular mapping catheter. Secondary endpoints included (a) procedural parameters and (b) procedure‐related major complications, defined as transient ischemic attack (TIA), stroke, PN paralysis, pericardial tamponade, or severe bleeding requiring blood transfusion, and hematoma at the access site requiring surgical intervention.

### 2.6 Statistical analysis

Continuous data were summarized as means ± SD or as medians (25th and 75th percentiles) as appropriate. Categorical data were presented as N (%). Based on a logistic regression model (global test of no regression), baseline variables were simultaneously compared between treatment groups. Baseline data were age, gender, AF type, congestive heart failure, hypertension, diabetes mellitus, previous stroke/TIA, myocardial infarction, ejection fraction, and LA diameter.

Differences in procedural data (fluoroscopy dose and time, procedure time, and contrast media) were analyzed with Wilcoxon Mann‐Whitney tests. Differences between PV data were examined using linear and generalized linear mixed effects models. Multiple comparison procedures were applied to compare one toward all other PVs. PV data were the duration of freeze cycles, the minimal temperature, the temperature at the time of isolation, TTI, the number of freeze cycles, and the isolation of each vein.

All *P* values were two‐sided and a *P* value of <.05 was considered significant. All calculations were performed with the statistical analysis software R (R Core Team, 2019).

## 3 RESULTS

### 3.1 Patient characteristics

A total of 200 consecutive patients were included into this two‐center analysis. In group I (n = 100), AF at baseline was paroxysmal in 62 of 100 (62%) patients, and persistent in 38 of 100 (38%) patients. Median age was 55.5 (57, 73) years and mean LA diameter was 45 ± 6 mm, and 40 of 100 (40%) patients were female. Detailed patient data and the characteristics of the control group (n = 100) are given in Table 1. There was no significant difference regarding baseline data between both groups (*P* = .31).

**Table 1:** Patients baseline characteristics

	Statistics	
Variable	Group I	Group II
Age (y)	55.5 (57, 73)	55.5 (57, 72)
Female	40 (40)	33 (33)
Left atrial diameter (mm)	44.7 ± 6	43.4 ± 5
Ejection fraction (%)	58.1 ± 6	58.8 ± 2
Type of atrial fibrillation		
Paroxysmal	62 (62)	72 (72)
Persistent	38 (38)	28 (28)
Congestive heart failure	7 (7)	1 (1)
Arterial hypertension	65 (65)	49 (49)
Diabetes mellitus	14 (14)	7 (7)
History of stroke/transient ischemic attack	5 (5)	3 (3)
History of myocardial infarction	6 (6)	11 (11)
CHA_2_DS_2_‐VASc score		
0	17 (17)	17 (17)
1	17 (17)	20 (20)
2	26 (26)	30 (30)
3	22 (22)	19 (19)
4	14 (14)	12 (12)
5	3 (3)	2 (2)
6	1 (1)	—

*Note*: Continuous data are summarized as means ± SD or as medians (25th and 75th percentiles). Categorical data are presented as n (%). Test of no regression (*P* = .31).

### 3.2 Procedural parameters and acute ablation results

Acute PVI was achieved in all patients of both groups. There was a statistically significant difference regarding median procedure time (group I: 65 [55, 82.5] vs group II: 82.5 [65, 105] minutes, *P* < .001), whereas median fluoroscopy duration (group I: 14.2 [11.8, 20.2] vs group II: 14.2 [12, 20] minutes; *P* = .99) and dosage (group I: 822 [450, 1350] vs group II: 970 [563, 1869] cGy cm^2^, *P* = .27) were comparable for both groups.

A total of 793 PVs were identified and successfully isolated (200 right superior PVs, 200 right inferior PVs [RIPV], 193 left superior PVs, 193 left inferior PVs, and seven left common PVs).

The overall mean freeze cycle duration was 175 ± 35 seconds and significantly shorter in group I (group I: 166 ± 29 vs group II: 183 ± 28 seconds, *P* < .001). There was no statistically significant difference regarding the overall mean minimal CB temperature (group I: −46.0 ± 6°C vs group II: −46.1 ± 6°C, *P* = .84). Lowest overall endoluminal esophageal mean temperature was 25 ± 9°C. In no case, the cryoapplication had to be stopped due to endoluminal esophageal temperatures below the cutoff value of 15°C. Procedural details are given in Table 2.

**Table 2:** Procedural data

	Statistics		
Variable	Group I	Group II	*P* value
Mean minimal temperature (°C)	−46.1 ± 6	−46.0 ± 5.9	.84
Mean time to PVI (s)	33 (23, 50)	40 (26, 60)	<.01
Mean temperature at TTI (°C)	−28.2 ± 9.6	−32.2 ± 8.8	.18
Mean total freezing duration (s)	165.5 ± 29	183.2 ± 38	<.01
Total procedure time (min)	65 (55, 82.5)	82.5 (65, 105)	<.01
Total fluoroscopy time (min)	14.2 (11.8, 20.2)	14.2 (12, 20)	.99
Total fluoroscopy dosage (cGy cm^2^)	822 (450, 1350)	970 (563, 1869)	.27
Contrast medium (mL)	122 ± 30	117 ± 33	.56

*Note*: Continuous data are summarized as means ± SD or as medians (25th and 75th percentiles). Categorical data are presented as n (%).

Abbreviations: PVI, pulmonary vein isolation; TTI, time to isolation.

### 3.3 Real‐time PV recordings

**Figure 3:**
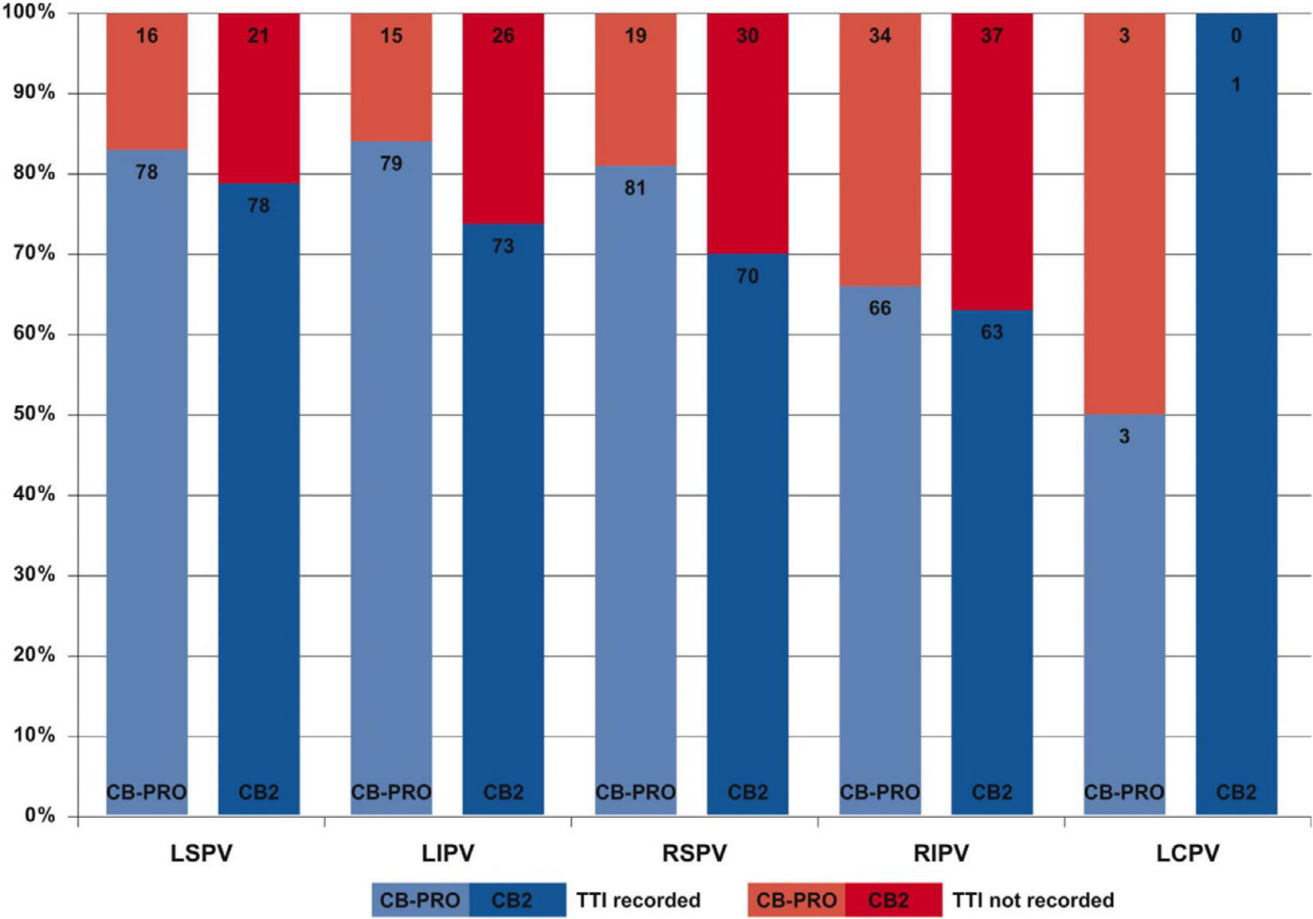
The verification of PVI using the novel CB‐Advance PRO in comparison to the TTI data of patients ablated using the CB2 is summarized. CB, cryoballoon; CB2, second‐generation CB; CB‐Advance PRO, fourth‐generation CB; LCPV, left common PV; LIPV, left inferior PV; LSPV, left superior PV; PV, pulmonary vein; PVI, PV isolation; RIPV, right inferior PV; RSPV, right superior PV; TTI, time to isolation

There was a trend toward a higher rate of online registration of PV signals in group I as compared to group II (77.9% vs 71.4%) not reaching statistical significance (*P* = .09). Overall, for the RIPV, TTI was recorded significantly less often when compared to all other PVs (*P* < .001). There was no statistically significant difference between both groups regarding the rate of TTI per PV (*P* = .56). Median TTI was significantly shorter when using the CB‐Advanced PRO (group I: 33 [23, 50] vs group II: 40 [26, 60] seconds, *P* < .01). Ablation data per individual PV is depicted in Figure 3.

### 3.4 Periprocedural complications

A total of 3 of 100 (3%) patients in group I and 1 of 100 patients (1%) in group II suffered from a transient PN paralysis during energy delivery along the right pulmonary vein (RPVs). The PN in all four patients fully recovered during the procedure. No further complications occurred. There was no statistically significant difference regarding periprocedural complications (*P* = .62) between both groups.

## 4 DISCUSSION

### 4.1 Main findings

The present study is the first to report on feasibility, efficacy, and safety of catheter ablation for symptomatic AF using the novel CB‐Advance PRO as compared to patients treated with the CB2.

The main findings are:

1. The CB‐PRO provides a high rate of acute PVI during the index freeze cycle.
2. There was no statistically significant difference regarding the incidence of PVs with TTI between the CB‐Advance PRO and the CB2.
3. Median TTI and mean total freezing time were significantly shorter when using the CB‐Advance PRO as compared to the CB2.

### 4.2 Acute efficacy of CB‐based PVI

While RF‐based PVI was considered the “gold standard” for the treatment of AF for a long time, the CB2 has turned out to be an effective alternative for AF ablation.^1^, ^12^, ^13^ The FIRE AND ICE trial, which prospectively randomized PAF patients to either RF or CB‐based PVI, established the noninferiority of CB ablation vs RF ablation in terms of efficacy and safety.^6^ As PVI is the first‐line recommended procedural endpoint in AF ablation, it can be recommended not only for the treatment of PAF but also for persistent AF, as studies using CB in patients with paroxysmal and even with persistent AF have shown a comparable clinical success rate.^14^, ^15^, ^16^, ^17^, ^18^ Today, CB‐based PVI is an accepted strategy for the treatment of symptomatic, drug‐refractory AF and is therefore recommended by the current AF ablation guidelines.^1^ Thus, the CB turned out as a tool, which is increasingly utilized in many electrophysiology (EP) laboratories worldwide.

### 4.3 CB‐based PVI—Can we still do better?

The design of the CB has been revised several times—inter alia to improve cooling properties and visualization rates of real‐time PVI.^10^, ^19^ Compared to the first‐generation CB (CB1), the CB2 provides a more uniform and distal cooling and therefore permits balloon angulations for challenging PV anatomy without leaving the optimal freezing zone.^19^ Although the CB1 had several limitations, clinical results when using the further generation CBs have been shown to be comparable to RF.^6^ The initially most commonly applied ablation protocol has been the bonus‐freeze protocol characterized by an additional empiric freeze cycle following documentation of electrical PVI.^20^, ^21^ Although the current trend is to perform CB ablations without an additional bonus freeze, the optimal dosing strategy in terms of duration of cryoapplication per freeze is not yet established. 'TTI‐guided ablation has been proposed to be superior with regards to procedure time, freeze cycle duration, and PVI durability depending on bonus‐freeze application strategy and in some studies was associated with beneficial clinical outcomes.^8^, ^9^, ^22^ For a TTI‐guided approach, a visualization of real‐time recordings of PV signals is mandatory. Thus, it is of major interest to optimize the catheter design of the CB for a potentially higher rate of real‐time recordings. The design of the third‐generation CB (CB3) was quite similar to the CB2 except for a shortened tip and already promised higher rate of TTI,^10^, ^23^ but was taken from the European market, because of observed differences in minimal CB freeze temperatures most likely due to a changed position of the temperature probe of the CB3 to a more proximal position, which causes a minimally longer distance of the returning gas to the temperature probe and therefore higher returning gas temperatures.

Lately, the novel CB‐Advance PRO was designed to enhance the probability of real‐time recordings due to a 40% shortened catheter tip and to optimize catheter maneuvering, whereas the technical properties of the balloon itself remained mainly unchanged when compared to the CB2, especially regarding cooling properties.

The present study sought to evaluate, whether there is an increased rate of PV real‐time recordings and TTI when using the CB‐Advance PRO as compared to the widely used CB2.

The present study confirms that the feasibility and safety of the CB‐Advance PRO is comparable to the CB2. In our analysis, all PVs were successfully isolated. It was demonstrated that the incidence of major or minor complications and in particular the incidence of PN palsy was similar in both patient groups.

In our analysis, a strong numerical trend toward a higher incidence of real‐time recordings of the PVs using the novel CB‐Advance PRO when compared to a control group of patients treated with the CB2 was seen (78% vs 71%), although no statistical significance could be reached. In addition, total mean freezing duration time (group I: 166 ± 29 vs group II: 183 ± 28 seconds, *P* < .001) and median TTI were significantly shorter when using the CB‐Advance PRO (group I: 33 [23, 50] vs group II: 40 [26, 60] seconds, *P* < .01). The proximity of the mapping catheter due to the shortened tip of the CB may provide a better measurement of the signals inside the PVs and helps the operator to assess the achievement of PVI earlier. The improved procedure times might be explained by an optimized balloon position due to the shorter tip of the balloon. Further studies have to confirm these findings. Furthermore, the results of our study have to be interpreted against the background, that all operators involved into the procedures were physicians highly experienced with CB ablation. Experienced operators can easily perform multiple maneuvers in order to get the spiral mapping catheter in a proximal PV position and to increase the visualization rate of real‐time PV recordings. However, less experienced operators will profit from the shortened CB tip which facilitates and simplifies proximal spiral mapping catheter positions.

## 5 LIMITATIONS

The current study is a retrospective analysis involving typical limitations. As this is not a randomized controlled study, any conclusions are hypothesis‐generating only. Nonetheless, to the best of our knowledge, this multicenter study is the first to describe the feasibility, acute efficacy, and safety of AF ablation using the novel CB‐Advance PRO in comparison to the CB2.

## 6 CONCLUSION

Catheter ablation of AF using the CB‐Advance PRO is feasible and safe and is associated with encouraging acute clinical success rate. Regarding the rate of real‐time PV recordings, it is comparable to the CB2 but associated with a significant reduction in mean TTI and mean total freezing time as compared to the CB2.

## CONFLICT OF INTEREST

Karl‐Heinz Kuck received travel grants and research grants from Biosense Webster, Stereotaxis, Prorhythm, Medtronic, Edwards, and Cryocath, and is a consultant to St. Jude Medical, Biosense Webster, Prorhythm, and Stereotaxis. He received speaker's honoraria from Medtronic. Andreas Metzner received speaker's honoraria and travel grants from Medtronic, Biosense Webster, and Cardiofocus. Shibu Mathew received speaker's honoraria and travel grants from Medtronic and Biosense Webster. Andreas Rillig received travel grants from Biosense, Medtronic, St. Jude Medical, Cardiofocus, EP Solutions, and Ablamap, and lecture and consultant fees from St. Jude Medical, Medtronic, Biosense, Cardiofocus, Novartis, and Boehringer Ingelheim.
